# Evaluating Nanotrap Microbiome Particles as A Wastewater Viral Concentration Method

**DOI:** 10.1007/s12560-024-09628-w

**Published:** 2025-01-04

**Authors:** Marlee Shaffer, Devin North, Kyle Bibby

**Affiliations:** Department of Civil and Environmental Engineering and Earth Sciences, University of Notre Dame, Notre Dame, IN 46556 USA

**Keywords:** Viral concentration, Wastewater-based epidemiology, Metagenome, Virome, Nanotrap Microbiome Particles

## Abstract

Wastewater-based surveillance has emerged as a powerful approach to monitoring infectious diseases within a community. Typically, wastewater samples are concentrated before viral analyses to improve sensitivity. Current concentration methods vary in time requirements, costs, and efficiency. Here, we evaluated the concentration efficiency and bias of a novel viral concentration approach, Nanotrap Microbiome Particles (NMP), in wastewater. NMP concentration efficiency was target-specific, with significantly lower concentrations of the bacterial indicator HF183 and viral indicator *Carjivirus* (formerly crAssphage) relative to direct extraction (1.2 × 10^5^ vs. 3.4 × 10^5^ GC/mL and 2.0 × 10^5^ vs. 1.2 × 10^5^ GC/mL, respectively), but significantly higher concentrations of the viral fecal indicator Pepper Mild Mottle Virus (PMMoV) relative to direct extraction (1.4 × 10^5^ vs. 8.4 × 10^3^ GC/mL). Targeted metagenomic sequencing showed that NMP resulted in significantly more unique species reads per sample than direct extractions (*p* < 0.001) by detecting species that went undetected by direct extractions. Key viral families identified with high abundances were Adenoviridae, Caliciviridae, Herpesviridae, Papillomaviridae, and Polyomaviridae. NMP showed differential ability for concentrating clinically relevant viral families, suggesting that the technology should be evaluated and optimized for specific viral targets before implementation.

**Supplementary Information:**

The online version contains supplementary material available at 10.1007/s12560-024-09628-w.

## Introduction

The use of wastewater-based epidemiology (WBE) to track diseases of interest and inform public health decisions has dramatically increased since the start of the COVID-19 pandemic (Barcellos et al., 2023). Individuals infected with enteric or respiratory viruses can shed high quantities of viral particles in their feces, urine, sputum, or saliva, which ultimately collects in the wastewater stream (Chen & Bibby, 2023a, 2023b; Crank et al., 2022; G. La Rosa et al., 2012). WBE can provide rapid and reliable information about diseases circulating in a community, enabling public health officials to respond and adapt interventions to mitigate outbreaks (Mao et al., 2020). WBE is advantageous for tracking viral diseases due to community-level monitoring, non-invasive, anonymous techniques, and the ability to capture asymptomatic cases, unlike clinical surveillance (Barcellos et al., 2023; Chen and Bibby 2023c; Mao et al., 2020).

However, monitoring viral pathogens in wastewater poses challenges due to typically low viral concentrations in sewage (North & Bibby, 2023), necessitating sample concentration prior to analysis. Current concentration methods vary in efficiency, costs, and time requirements (North & Bibby, 2023). Faster concentration and improved isolation of viruses can improve turnaround times, thereby better informing public health decisions.

Nanotrap Microbiome Particles (NMP) are a novel proprietary microbial concentration technology designed to rapidly capture and concentrate microorganisms. NMP utilize chemical affinity baits to bind microbes of interest to porous hydrogel particles (Ceres Nano 2022). The exact mechanism for viral capture is unknown, though it is hypothesized that glycoproteins on the virion interact with NMP (Lin et al., 2020). Without a defined mechanism of viral capture, biases may be introduced due to varying concentration efficacy of target microbes. Research is needed to understand those biases and their implications of using this concentration method for WBE or sequencing experiments.

NMP have demonstrated the ability to capture and concentrate a wide range of viruses, including human immunodeficiency virus, Rift Valley fever virus, influenza viruses, Zika virus, Dengue, chikungunya, SARS-CoV-2, and Respiratory Syncytial Virus in wastewater and clinical samples (Andersen et al., 2023; Lin et al., 2020). NMP concentrated viral RNA in clinical urine samples by at least one log_10_ compared to samples without NMP, resulting in more reliable RT-qPCR results (Lin et al., 2020). When analyzing the concentrations of SARS-CoV-2 in wastewater samples, the efficiency of detection increased by almost twofold with NMP compared to a direct extraction (Andersen et al., 2023). Furthermore, the use of NMP can be advantageous during process automation as protocols have been extensively produced, further improving turnaround times.

Defining NMP’s potential biases as a concentration method is critical to inform its application in WBE. In this study, we comprehensively evaluate NMP target concentration in wastewater using both targeted digital PCR and metagenomic sequencing.

## Materials and Methods

### Wastewater Collection

We collected eight primary influent wastewater (1 L) samples aseptically from an anonymous wastewater treatment plant in Northern Indiana over six weeks. Samples were processed immediately upon collection. For each sample, the wastewater was evaluated for chemical oxygen demand (COD), total nitrogen, total suspended solids (TSS), and pH following EPA and manufacturer's protocols (Table S1).

### Nanotrap Concentration

Nanotrap Microbiome A Particles (NMP) (Ceres Nanosciences, Manassas, VA) were used on three aliquots for each sample following the manufacturer's protocol (Ceres Nano n.d.). Briefly, 10 mL of homogeneous primary influent wastewater was aseptically transferred to a 15-mL Falcon tube, and 100 µL of Nanotrap Enhancement Reagent 2 (ER2) and 150 µL of NMP were added. Samples were inverted three times and incubated at room temperature (25 °C) for 10 min before magnetizing (DynaMag-15 Magnet) to separate the particles from the solution. The supernatant was removed without disturbing the pellet, and 1 mL of molecular-grade water was used to resuspend the particles before transferring the solution to a 2-mL tube. The tube was magnetized (DynaMag-2 Magnet), and the supernatant was discarded. The pellet was resuspended with 500 µL of PM-1 (Qiagen) and the resulting solution was incubated at 95 °C for 10 min. NMP were separated using a magnetic rack, and the supernatant was used downstream for extraction.

### Nucleic Acid Extraction

Three 500 µL aliquots of homogenized primary influent wastewater were used for each sample collected. Each aliquot was placed in a Glass Bead Beating tube, and 600 µL of PM1 and 6 µL of beta-mercaptoethanol were added. Each tube was homogenized using an MP Bio Fast-Prep 24 for four rounds of 20 s at 4 m/s. Qiagen AllPrep PowerViral DNA/RNA Kit was then used per the manufacturer's protocol. For NMP samples, the nucleic acids were extracted using the Qiagen AllPrep PowerViral DNA/RNA Kit and the procedure from Ceres Nanosciences.

### dPCR Quantification

HF183, *Carjivirus* (formerly crAssphage), and Pepper Mild Mottle Virus (PMMoV) concentrations were assessed using the QIAcuity digital PCR System (Green et al., 2014; Haramoto et al., 2013; Stachler et al., 2017; Turner et al., 2023). Primer and probe sequences, master mix solutions, and thermocycling conditions are detailed in Table S2. The QIAcuity Probe PCR Kit was used for HF183 and *Carjivirus* quantification and the QIAcuity One-Step Viral RT-PCR Kit was used for PMMoV quantification following the manufacturer’s protocol. Each well had 2 µL of samples added along with 12 µL of master mix. The solutions were vortexed before transferring 12 µL to the 8.5 k, 96-well Nanoplate. Each nanoplate contained no template and negative extraction controls for DNA and RNA targets. All data were analyzed using the QIAcuity Suite Software, and manual thresholds were used to determine negative and positive partitions. Raw concentration values were normalized for influent wastewater volumes using the following Eq. 1.

1 $$C= \frac{R x {V}_{r} x {V}_{e} x D}{{V}_{t }x {V}_{s}},$$

where

C is the calculated concentration (genome copies/mL).

R is the raw concentration (genome copies/µL).

V_r_ is the reaction volume (µL).

V_e_ is the elution volume (µL).

V_t_ is the template volume (µL).

V_s_ is the sample volume (mL).

D is the dilution factor, if applicable.

### Sequencing

Before library preparation, reverse transcription was conducted using SuperScript™ IV Reverse Transcriptase per the manufacturer's protocol. Libraries were then prepared using the xGen ssDNA & Low-Input DNA Library Preparation Kit with Normalase and UDI Primers Plate 1 following the manufacturer’s instructions. Finished libraries were sent to Azenta Life Sciences (New Jersey, USA) for sequencing on the Illumina HiSeq with 2 × 150 bp configuration.

QIAseq® xHYB Viral and Bacterial Library Kit with the Adventitious Agent Panel was used for a second library preparation following the manufacturer’s protocol using the DNA/RNA extraction. The QIAseq® xHYB Adventitious Agent Panel enriches for 132 viral targets. Finished barcoded libraries were sent to Azenta Life Sciences for sequencing with the Illumina HiSeq with 2 × 150 bp configuration. Data were deposited to the Sequence Read Archive (Accession Number: PRJNA1113795).

### Statistical Analysis

We performed all statistical analysis and graphs with R Studio 2023.09.0 (RStudio Team n.d.). Wilcoxon signed-rank tests were used to determine the statistical significance of dPCR results and the abundance data for the sequencing panel. KBase was used to analyze the initial sequencing using Kaiju v1.9.0 to classify the taxonomy of metagenomic reads (Chivian et al., 2023; Menzel et al., 2016). Results from the Qiagen Library Prep Kit were submitted to GeneGlobe (QIAGEN) for further analysis.

## Results and Discussion

### Quantification of DNA and RNA targets using dPCR

**Fig. 1:**
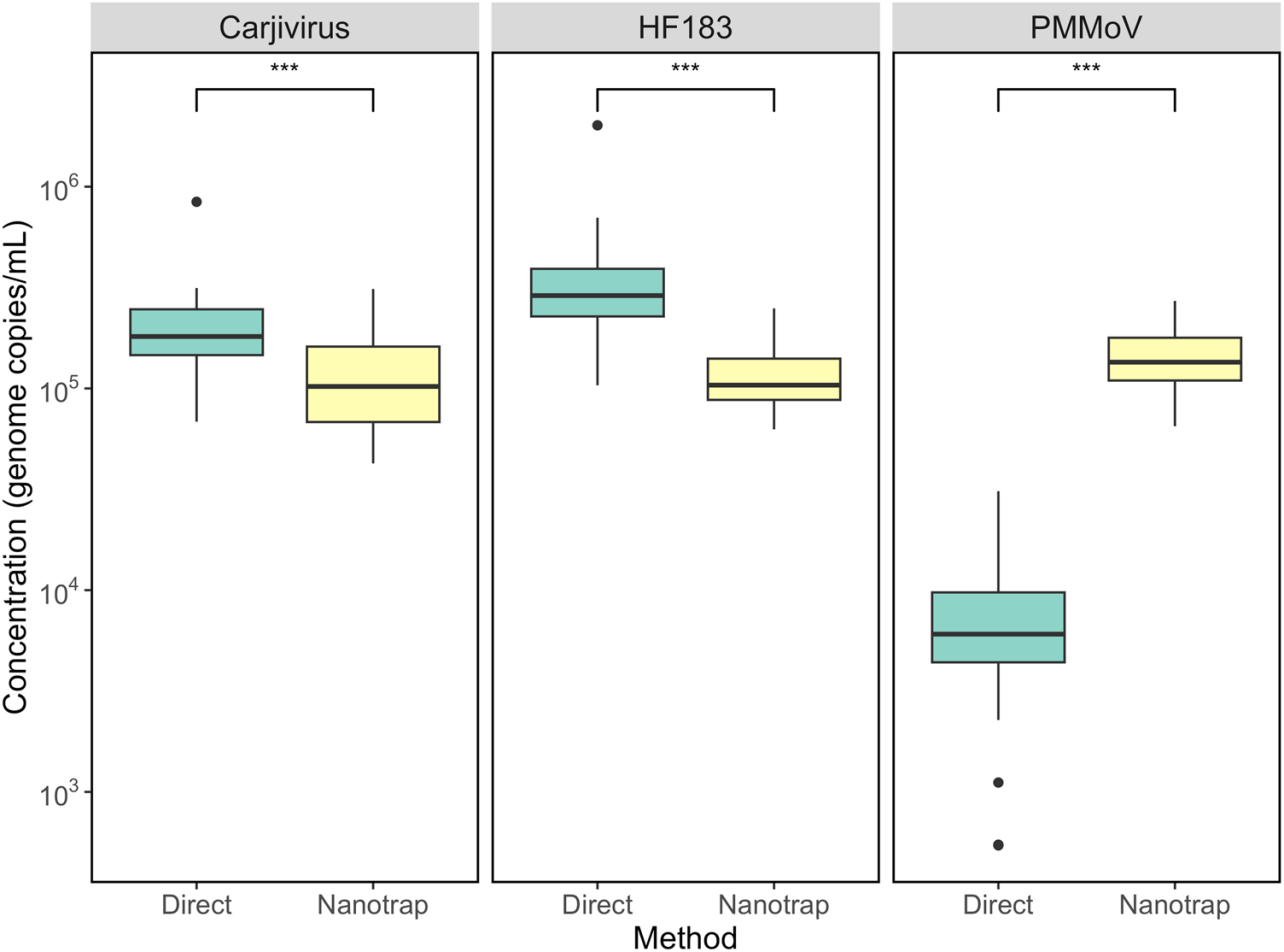
dPCR Comparison of *Carjivirus*, HF183, and PMMoV for NMP and Direct Extraction Methods. A Wilcoxon test was used for statistical comparison, resulting in a p value < 0.001 for each target. The black circles represent outliers in the dPCR data

We collected eight primary influent wastewater samples and conducted three experimental replicates for both NMP and direct extraction methods. Each sample was analyzed using two digital PCR (dPCR) replicates for each target of interest. Figure 1 shows the results for three targets: *Carjivirus*, HF183, and Pepper Mild Mottle Virus (PMMoV). These targets are commonly used human fecal indicators and are used as normalization markers signifying their importance for WBE initiatives and water quality assessments (Eifan et al., 2023; Farkas et al., 2019; Green et al., 2014).

The use of NMP showed target-specific concentration effects for *Carjivirus*, HF183, and PMMoV in primary influent wastewater samples. Specifically, NMP resulted in significantly lower concentrations (both with p < 0.001) of HF183 and *Carjivirus* compared to direct extractions (1.2 × 10^5^ vs. 3.4 × 10^5^ GC/mL and 2.0 × 10^5^ vs. 1.2 × 10^5^ GC/mL, respectively). Conversely, PMMoV concentrations were significantly higher (p < 0.001) with NMP compared to direct extractions (1.4 × 10^5^ vs. 8.4 × 10^3^ GC/mL). These results highlight the target-specific concentration effects of NMP as *Carjivirus* and PMMoV exhibited differential concentration effects relative to direct extraction in primary influent wastewater.

Our results are consistent with other concentration studies that have demonstrated differential concentration effects for various targets. For instance, a previous study comparing multiple concentration methods reported higher concentrations of *Carjivirus* using direct extractions compared to polyethylene glycol (PEG) precipitation, skimmed milk flocculation, pH drop, and Amicon ultrafiltration concentration methods (North & Bibby, 2023). Previous studies found that *Carjivirus* had higher concentrations with an adsorption-extraction method and PEG methods compared to NMP, which is agreeable with the results in this study (Ahmed et al., 2023a, 2023b; Farkas et al., 2024). A study examining SARS-CoV-2 and PMMoV found that NMP achieved higher concentrations of PMMoV in 58.3% of samples compared to the absorption-extraction method (Ahmed et al., 2023a, 2023b). Fani et al. found that HF183 concentration were, on average, one order of magnitude higher than PMMoV with NMP (Fani et al., 2024), where we found that HF183 and PMMoV concentrations were similar for NMP and HF183 was approximately 1.5 log higher than PMMoV with direct extractions. Additionally, another study observed significantly higher concentrations of SARS-CoV-2 when using NMP during conventional PCR, indicating the method’s successful concentration for specific viruses of interest (Andersen et al., 2023). This point was furthered by Ahmed et al. where NMP had differential concentration affinity compared to adsorption-extraction method for enteric and respiratory viruses (Ahmed et al., 2023a, 2023b), indicating specific targets should be assessed for use with NMP.

### NMP Shotgun Metagenomic Sequencing

Extracted direct and NMP samples were initially processed for shotgun metagenomic sequencing. Metagenomics sequencing offers an unbiased approach to sequence genomic material in a sample, but for viruses, a relatively small amount of sequences are known, and there are a variety of structures and genomes (Hjelmsø et al., 2017). Shotgun metagenomic sequencing resulted in a significant fraction of bacterial sequences (Figure S1). For NMP and direct extractions, 98.85% and 99.14% of reads were bacterial. Bacterial targets are commonly identified at high percentages in metagenomic studies due to the larger size of bacterial genomes compared to viral genomes and the high abundance of bacteria in wastewater (Cantalupo et al., 2011; Child et al., 2023; Tisza et al., 2023). For NMP and direct extractions, only 0.26% and 0.24% of reads, respectively, were viral, indicating that NMP did not enrich the viral fraction in wastewater. When these sequences were analyzed for *Carjivirus* or PMMoV, there were no identified reads, even though dPCR results showed they were present in all samples in high concentrations (Fig. 1). These results suggest that alternative methods are necessary to enrich the viral fraction when using NMP if untargeted analysis approaches, such as shotgun metagenomics, are to be used.

**Fig. 2:**
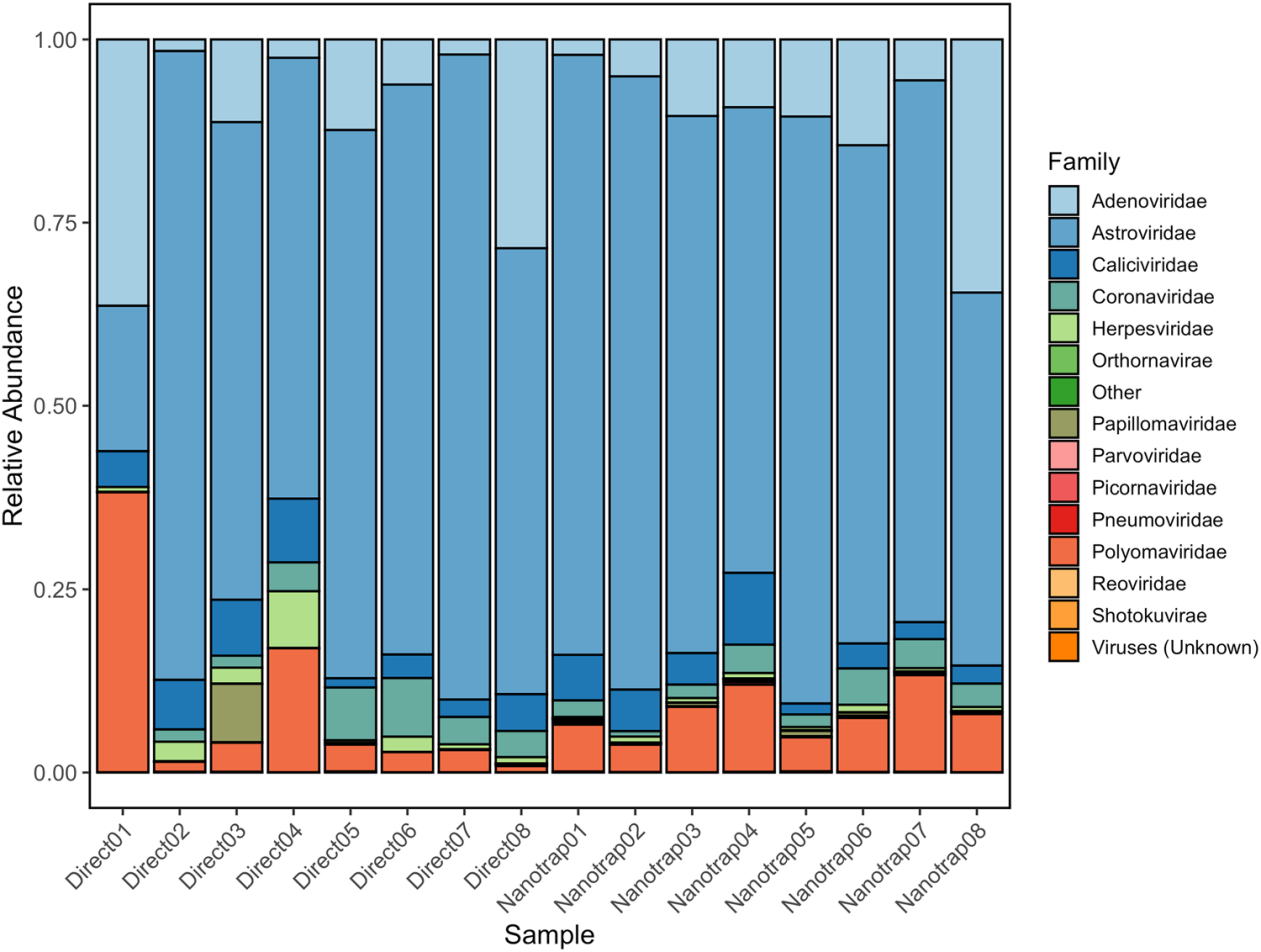
Selected family relative abundances using adventitious agent panel with NMP and direct extraction methods. others represent families that are rare or non-human pathogenic

To better assess the viral diversity in wastewater, we subsequently used a targeted viral enrichment panel prior to sequencing. A similar pattern was observed in a recent study that employed shotgun metagenomics and a targeted respiratory panel with hybrid capture, demonstrating increased detection of respiratory viruses in wastewater when using the targeted panel (Child et al., 2023), indicating that targeted panels might be beneficial for WBE initiatives for pathogenic viruses of interest. As our aim was to demonstrate concentration efficiency and bias of viruses in wastewater with NMP, the targeted panel was selected which included 132 human pathogenic viral targets. Figure 2 shows the family-level relative abundance for selected viral families, while Figure S3 depicts the relative abundance of each family in each sample. Our results indicated that Astroviridae and Adenoviridae dominated both the NMP and direct extraction sequences. These findings align with previous studies, which indicated that Astroviridae is abundant in wastewater, likely because it is one of the leading causes of acute gastroenteritis in children (Meleg et al., 2006). Similarly, Adenoviruses are detected more frequently in sewage than other enteric viruses (Fong et al., 2010).

**Fig. 3:**
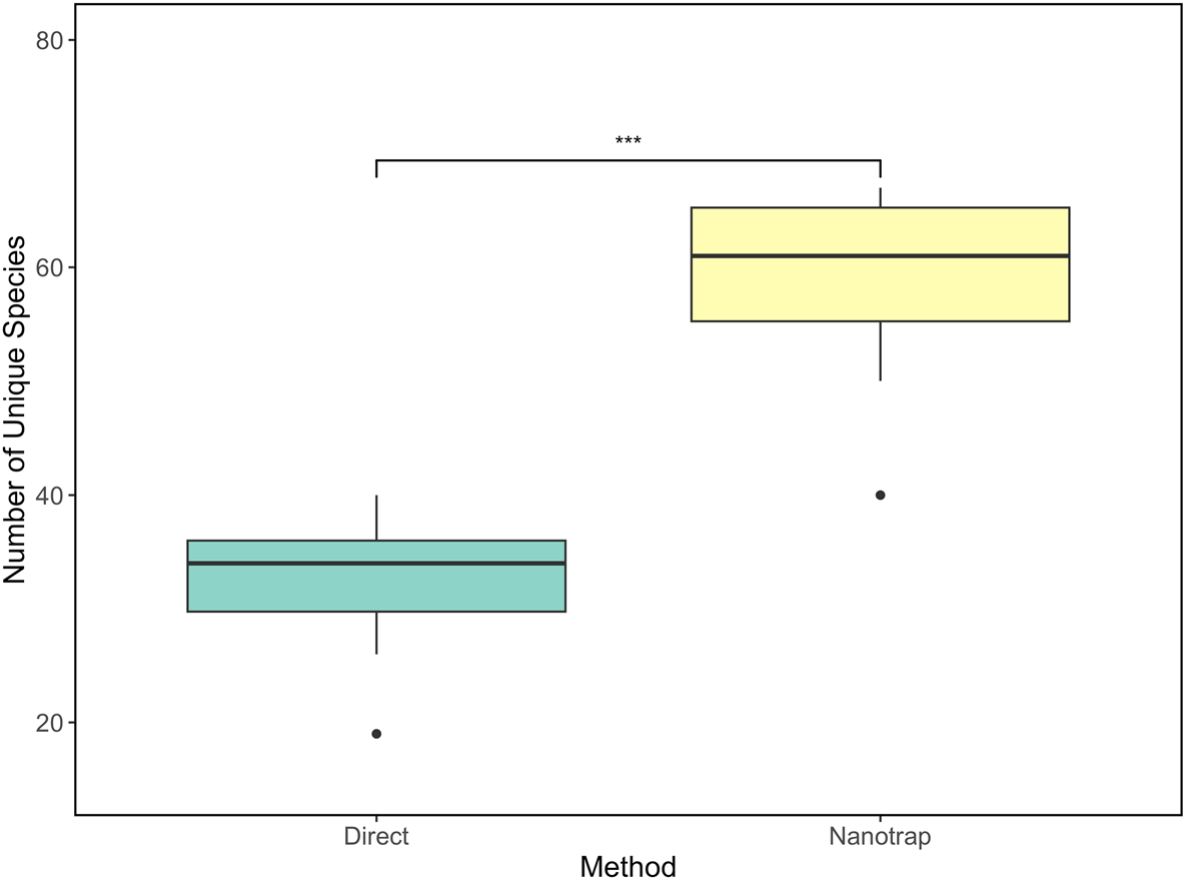
Alpha diversity of direct extraction and NMP for the targeted sequencing panel results. The boxplots show the mean and the 25 and 75 percentiles of the eight samples based on species-level viral reads. A Wilcoxon test was used for statistical comparison, resulting in a p value < 0.001

NMP showed significantly higher alpha diversity (p < 0.001) than the direct extraction method for the same samples when using the targeted enrichment panel (Fig. 3). The mean number of viral species was 32 (95% CI: 25.3 – 38.7) for direct extractions and 58 (95% CI: 49.1 – 67.65) for NMP. This increase in viral species using NMP suggests a greater efficiency for viral capture than direct extractions. NMP had a larger input volume compared to direct extraction, which could result in an increased sensitivity leading to a higher alpha diversity. These results underscore the inherent bias of NMP compared to a direct extraction method, indicating that certain viral families may have a higher affinity for concentration with NMP.

### Selected Family Sequencing Results

**Fig. 4:**
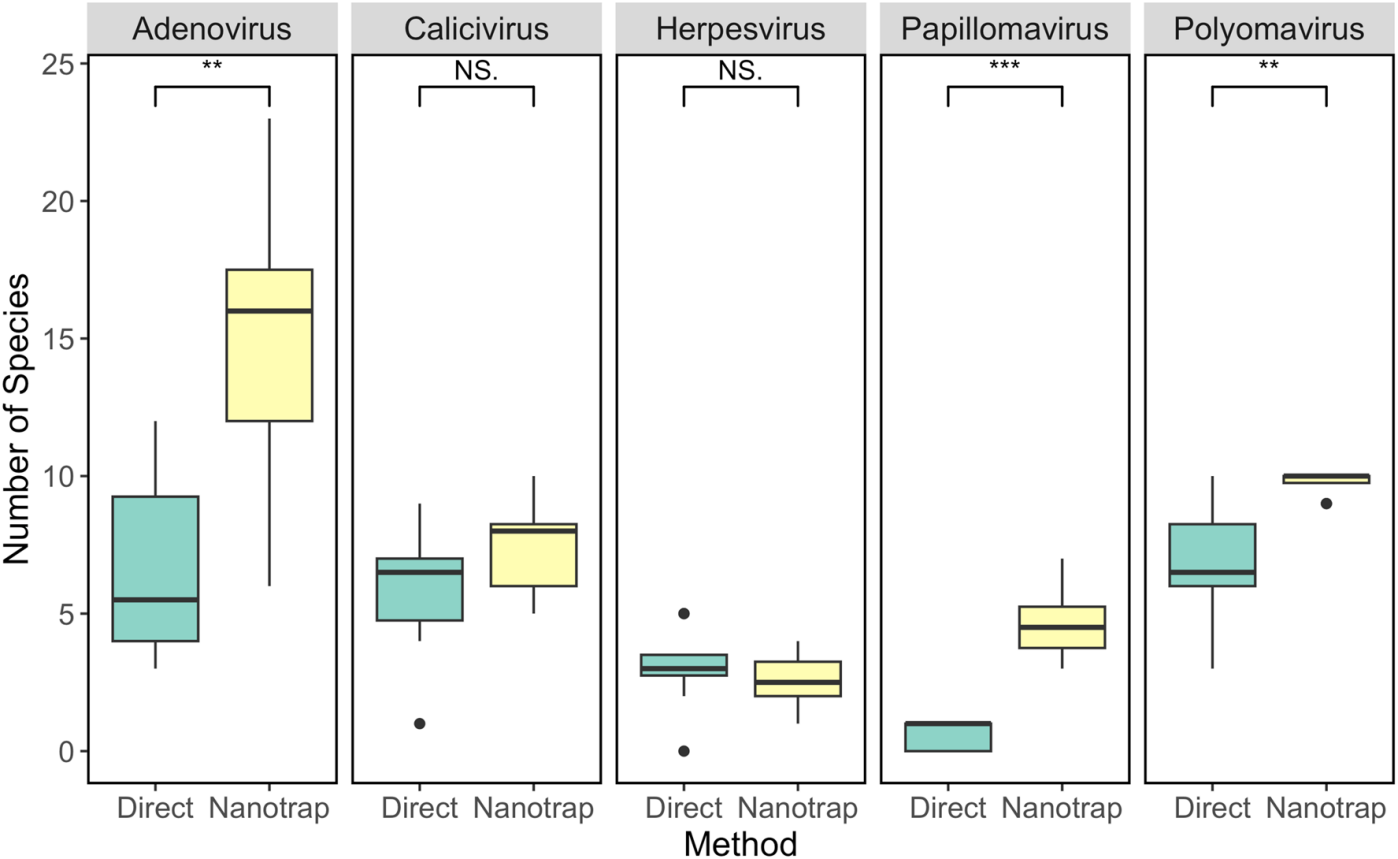
Alpha diversity of selected viral families for direct extraction and NMP. The boxplots show the mean and the 25 and 75 percentiles of the eight samples based on hits of species-level viral reads. A Wilcoxon test was used for statistical comparison

Five families of interest were selected for further analysis at the species level: Adenoviridae, Caliciviridae, Herpesviridae, Papillomaviridae, and Polyomaviridae. These viral families contain clinically relevant species and are shed in human excreta (La Rosa et al., 2013; Matrajt et al., 2017; Qiu et al., 2023; Vanchiere et al., 2005, 2009), suggesting their potential suitability for future WBE initiatives. Evaluating concentration techniques is crucial to ensure the desired targets are adequately concentrated before use in WBE pipelines. Targeted metagenomic sequencing with NMP and direct extractions revealed differential concentrations of viral targets (Fig. 4). NMP yielded significantly higher (p < 0.01) numbers of viral species for Adenoviridae, Papillomaviridae, and Polyomaviridae than direct extractions. These results suggest that NMP exhibit a concentration bias for specific viral families and may be more effective for targeting these viruses for optimal capture and concentration. Furthermore, these results align with another study which used NMP and a probe sequencing panel which consistently found Polyomaviridae, Adenoviridae, and Caliciviridae when using NMP workflows (Jiang et al., 2024). The increased viral species numbers observed with NMP can enhance WBE efforts by providing a more comprehensive representation of viral diversity within these specific viral families.

### Limitations and Implications

During this study, sampling was conducted at a single wastewater treatment plant, potentially limiting the ability to identify correlations with physiochemical parameters associated with different wastewater sources. Moreover, the different sample input quantities for NMP (10 mL) and direct extractions (0.5 mL) may have introduced greater sequencing diversity in NMP concentrated samples. More PCR inhibitors may have been present with a higher input concentration which could affect the cDNA synthesis. Future work should compare concentration methods using the same input volume of sample to determine the efficiency related to commonly used methods. Future studies should also determine the suitability of NMP for specific targets of interest before implementation.

Targeted sequencing is advantageous if the targets of interest are both sequenced previously and included on the panel, allowing for identification of specific viral families. These methods enhance sensitivity and specificity, enabling the detection of known pathogens even at low concentrations (Pei et al., 2023). Targeted sequencing streamlines workflow and bioinformatics analysis, thereby reducing turnaround time and computational power (Pei et al., 2023). However, targeted sequencing has limitations, including its potential to restrict the ability to observe the overall viral metagenome or the discovery of emergent pathogens. Additionally, targeted PCR amplification can skew results and lead to nonuniform target coverage (Pei et al., 2023). Careful consideration is necessary to determine if targeted sequencing is appropriate based on the experimental objectives of the study.

## Conclusions

We demonstrate that NMP significantly improved overall sequencing diversity when using a targeted human pathogenic panel, yet inconclusive results with dPCR and shotgun metagenomic sequencing. Due to the rapid, scalable, and simple workflow of NMP, this methodology could be advantageous in limited resource settings or where there is a need to determine the presence or absence with a faster turnaround time than other concentration methods (Farkas et al., 2024; Liu et al., 2023). However, NMP exhibited a bias toward certain viruses, suggesting that specific viral characteristics may enhance their capture. Several clinically relevant viral families showed improved detection with NMP compared to direct extractions. Implementing NMP concurrently with WBE initiatives for specific viruses could benefit public health assessments. Further analysis of the introduced biases should be considered to optimize the effectiveness of NMP for WBE.

## Supplementary Information

Below is the link to the electronic supplementary material.

Supplementary file1 (DOCX 12000 KB)

## Data Availability

Sequence data have been deposited in the Sequence Read Archive (Accession Number: PRJNA1113795).
