# Correction: Evaluating Nanotrap Microbiome Particles as A Wastewater Viral Concentration Method

**DOI:** 10.1007/s12560-026-09697-z

**Published:** 2026-05-21

**Authors:** Marlee Shaffer, Devin North, Kyle Bibby

**Affiliations:** Department of Civil and Environmental Engineering and Earth Sciences, University of Notre Dame, Notre Dame, IN 46556 USA

**Correction to: Food and Environmental Virology (2025) 17:10**

10.1007/s12560-024-09628-w

In this article, the declaration note was missing: “The authors declare the following competing financial interest(s): K.B. is a co-inventor on a patent entitled Cross-Assembly Phage DNA Sequences, Primers and Probes for PCR-based Identification of Human Fecal Pollution Sources (US Patent 12,258,639).”

This has now been corrected in the original publication of the article. The original article has been corrected.

