# Supplementary material for: Evaluating Nanotrap Microbiome Particles as A Wastewater Viral Concentration Method
Source: Food Environ Virol. 2025 Jan 4;17(1):10. doi: 10.1007/s12560-024-09628-w (PMC11700038; doi:10.1007/s12560-024-09628-w)
Supplement: Supplementary file 1 — Supplementary file1 (DOCX 12000 KB) [file 12560_2024_9628_MOESM1_ESM.docx]

**Supplementary Information: Evaluating Nanotrap Microbiome Particles as a Wastewater Viral Concentration Method**

Marlee Shaffer^a^ , Devin North ^a^, and Kyle Bibby^a*^

^a^Department of Civil and Environmental Engineering and Earth Sciences,

University of Notre Dame, IN 46556, USA

*Corresponding Authors: Kyle Bibby – [kbibby@nd.edu](mailto:kbibby@nd.edu)

5 pages, 2 Tables, 2 Figures

Table S1 – Wastewater Characterization

Table S2 – dPCR Primer and Probe Sequences and Thermocycling Conditions for CrAssphage, HF183, and PMMoV

Figure S1 – Metagenomic Low Abundance Sequencing Results

Figure S2 – Family Abundances from Adventitious Agent Panel

**Table S1.** Wastewater Characterization

|  | **COD (mg/L COD)** | **TN**  **(mg/L N)** | **TSS**  **(ppm)** | **pH** |
| --- | --- | --- | --- | --- |
| **Sample** | Method 8000 (Hach: HR COD Digestion Vials) | Method 10072 (HACH: TNT 40 HR Total Nitrogen with Persulfate Digestion) | Standard Methods 2540D |  |
| **01** | 113 | 30 | 110 | 6.68 |
| **02** | 161 | 28 | 100 | 7.49 |
| **03** | 175 | 43 | 90 | 7.52 |
| **04** | 198 | 66 | 110 | 6.81 |
| **05** | 214 | 64 | 130 | 7.02 |
| **06** | 177 | 32 | 90 | 6.74 |
| **07** | 224 | 17 | 80 | 7.54 |
| **08** | 243 | 45 | 120 | 7.04 |

**Table S2.** dPCR Primer and Probe Sequences and Thermocycling Conditions for *Carjivirus*, HF183, and PMMoV. All imaging used an exposure of 500 and a gain of 6.

| **Target** | **Primer and Probe Sequences** | **Thermocycling Conditions** | **Mastermix Solution** | **Ref** |
| --- | --- | --- | --- | --- |
| HF183 | F: ATCATGAGTTCACATGTCCG | 1 x 2 min at 95°C  40 x 15 sec at 95°C and 30 sec at 59°C | 3 µL of 4x Probe PCR Master Mix, 1.2 µL of primer-probe mix 3.9 µL of RNase-Free water | (1) |
|  | R: CTTCCTCTCAGAACCCCTATCC |  |  |  |
|  | P: (FAM)-CTAATGGAACGCATCCC-(MGB) |  |  |  |
| *Carjivirus* | F: CAGAAGTACAAACTCCTAAAAAACGTAGAG | 1 x 2 min at 95°C  40 x 15 sec at 95°C and 30 sec at 59°C |  | (2) |
|  | R: GATGACCAATAAACAAGCCATTAGC |  |  |  |
|  | P: (FAM)-AATAACGATTTACGTGATGTAAC-(MGB) |  |  |  |
| PMMoV | F: GAGTGGTTTGACCTTAACGTTTGA | 1 x 40 min at 50°C  1 x 2 min at 95°C  40 x 15 sec at 95°C and 30 sec at 59°C | 3 µL of 4x One-Step Viral RT-PCR Master Mix, 0.12 µL of 100x Multiplex Reverse Transcription Mix, 0.6 µL of 20x primer-probe mix, 7.2 µL of RNase-Free water | (3) |
|  | R: TTGTCGGTTGCAATGCAAGT |  |  |  |
|  | P: CCTACCGAAGCAAATG |  |  |  |

**
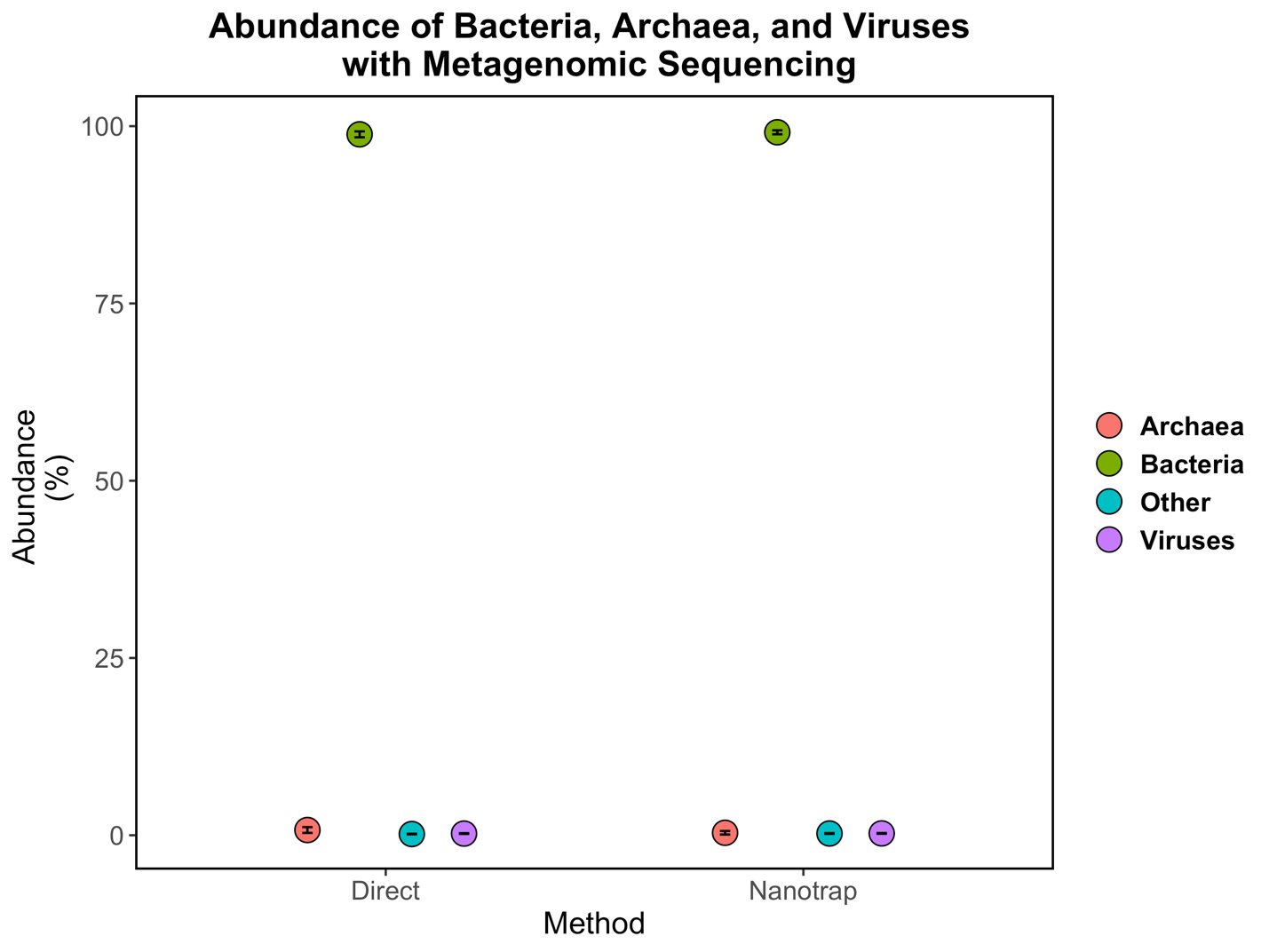
**

**Figure S1.** Metagenomic Low Abundance Sequencing Results. The y-axis shows the percent abundance of Archaea, Bacteria, Viruses, and Other from the initial metagenomic sequencing.

**
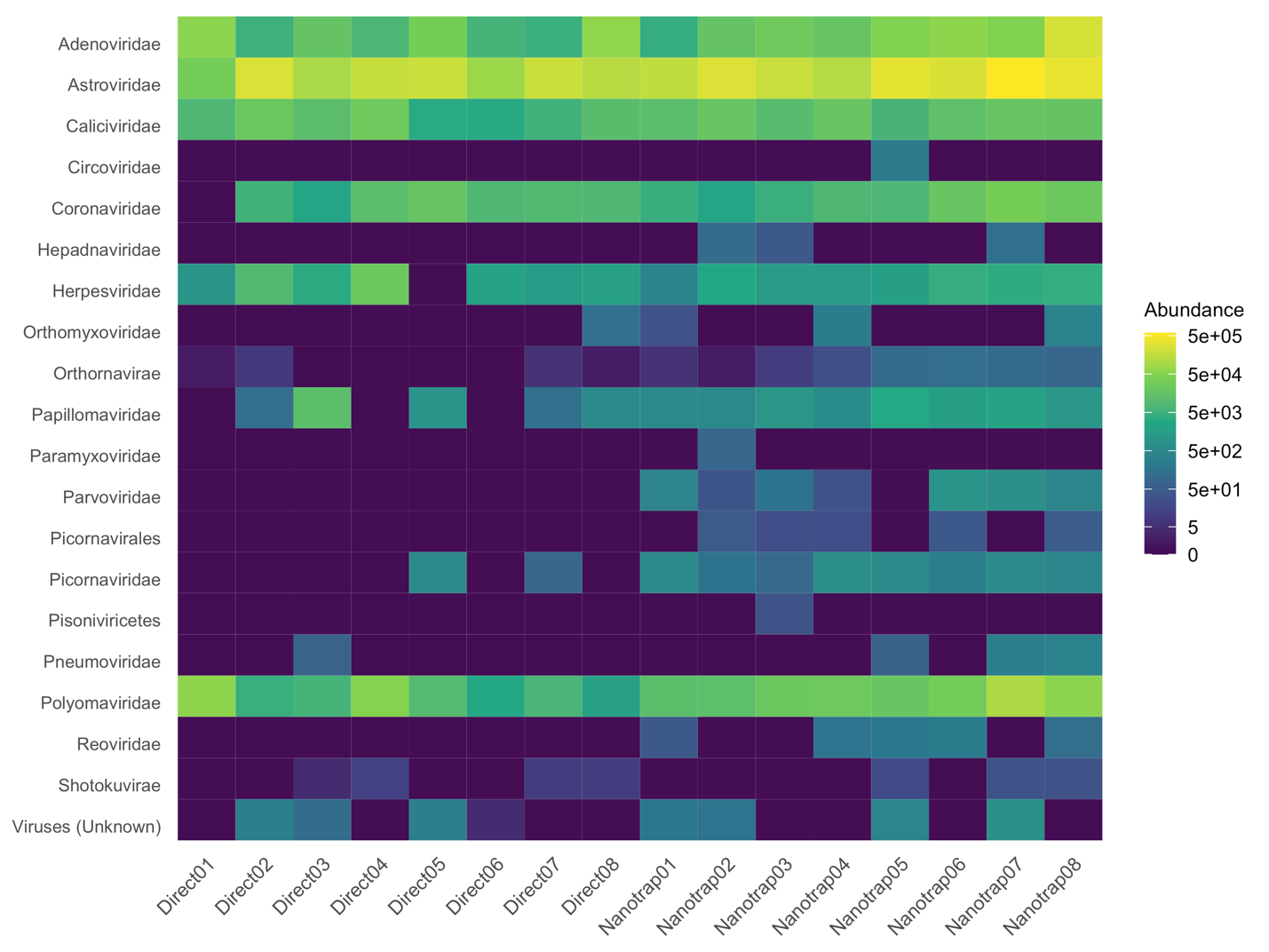
**

**Figure S2.** Family Abundances from Adventitious Agent Panel. All viral species at the family level are shown on the y-axis. The x-axis has the sample method and number, where the numbers correlate for both methods (i.e. they are from the same sample).

**References**

1. Green HC, Haugland RA, Varma M, Millen HT, Borchardt MA, Field KG, Walters WA, Knight R, Sivaganesan M, Kelty CA, Shanks OC. 2014. Improved HF183 Quantitative Real-Time PCR Assay for Characterization of Human Fecal Pollution in Ambient Surface Water Samples. Appl Environ Microbiol 80:3086–3094.

2. Stachler E, Kelty C, Sivaganesan M, Li X, Bibby K, Shanks OC. 2017. Quantitative CrAssphage PCR Assays for Human Fecal Pollution Measurement. Environ Sci Technol 51:9146–9154.

3. Haramoto E, Kitajima M, Kishida N, Konno Y, Katayama H, Asami M, Akiba M. 2013. Occurrence of Pepper Mild Mottle Virus in Drinking Water Sources in Japan. Appl Environ Microbiol 79:7413–7418.
